# Tongshu Capsule Down-Regulates the Expression of Estrogen Receptor α and Suppresses Human Breast Cancer Cell Proliferation

**DOI:** 10.1371/journal.pone.0104261

**Published:** 2014-08-07

**Authors:** Chengzhi Du, Zhen Li, Shuang Wang, Zhongmei Zhou, Jingkun Wang, Jian Dong, Ceshi Chen

**Affiliations:** 1 Key Laboratory of Animal Models and Human Disease Mechanisms of Chinese Academy of Sciences & Yunnan Province, Kunming Institute of Zoology, Kunming, Yunnan, China; 2 First Affiliated Hospital of Kunming Medical University, Kunming, Yunnan, China; 3 Yunnan Institute of Materia Medica, Kunming, Yunnan, China; South China Sun Yat-sen University Cancer Center, China

## Abstract

The Tongshu Capsule (TSC) is a prevalent form of traditional Chinese medicine widely used for its purported effects in treating mammary gland hyperplasia and inflammation. Though successful in several clinical studies, there is no clear evidence as to why TSC has a positive treatment effect, and little known about underlying mechanism that may account for it. In this study, we examined the effects of TSC and found that it has a comparatively strong growth inhibition on ERα positive breast cancer cells. TSC seems to cause G1 cell cycle arrest instead of apoptosis. Interestingly, TSC also down-regulated the expression of ERα and Cyclin D1. Consistently, TSC suppressed E2 mediated ERα downstream gene expression and cell proliferation in ERα positive breast cancer cell lines MCF7 and T47D. Depletion of ERα partially abolished the effects of TSC on the decrease of Cyclin D1 and cell viability. Our findings suggest that TSC may have therapeutic effects on ERα positive breast cancers and moreover that TSC may suppress breast epithelial cell proliferation by inhibiting the estrogen pathway.

## Introduction

Mammary hyperplasia and breast cancer are the most common breast diseases in women world-wide. Mammary hyperplasia itself is a benign hyperplasic disease that can develop into breast cancer. Both of these are primarily caused by over-proliferation of mammary epithelial cells resulting from hormone stimulation [1]. The accumulated evidence accordingly suggests that mammary hyperplasia and breast cancer are associated with ovarian hormone imbalance. Among patients with mammary hyperplasia, some studies have reported increased levels of estrogen and estrogen receptors [2]–[4]. High levels of serum estrogen are similarly associated with higher incidence of breast cancer [5], [6], and more than 60% of human breast tumors are positive for ERα. ERα participates in a variety of different signaling pathways in mammary epithelial cells [7], and promotes cell proliferation mainly via its regulation of G1-S cell cycle progression [8], [9]. Recent studies noting the successes of anti-estrogen treatments to patients with mammary hyperplasia or ERα-positive breast cancer [1], [10] supports that the estrogen signaling pathway plays an important role in the occurrence of mammary hyperplasia and breast cancer. Furthermore, the *CCND1 (CyclinD1)* oncogene is one of the major downstream transcriptional target genes of ERα in regulation of cell cycle progression [9], [11].

In the ongoing search for more effective therapeutic remedies to both breast cancer and mammary hyperplasia, there has been growing attention to non-traditional avenues of treatment options. In mainland China, for example, where the costs of typical treatments are often too expensive or unavailable, a popular option is to attempt treatment using traditional Chinese medicine. A popular form of traditional Chinese medicine for treating mammary hyperplasia and breast cancer is the Tongshu Capsules (TSC), developed by the Yi minority of Yunnan province. TSC is composed of eight traditional Chinese medicines, including notoginseng, scandent schefflera stem and leaf, erigeron breviscapus, delavay ampelopsis roos, rhizome panacismajoris, fructus gardeniae, Paris polyphylla, and licorice. In clinic, TSC is used to relieve mammary hyperplasia, swelling, pain, injuries, periarthritis, and gouty joints. Dr. Li, for example, used TSC to treat 60 patients with mammary hyperplasia, and was successful in around 80% of cases [12]. The result was further confirmed by a later independent study where Dr. Chen applied TSC combined with vitamin E to treat 112 patients with mammary hyperplasia with an effective rate of 97.32% [13].

Despite the promising results of these studies using TSC as a therapeutic, the underlying functional mechanisms of TSC are largely unknown. Likewise, these studies–though quite successful–only focused on treating mammary hyperplasia and to date TSC has not been tested to see if it is similarly effective in treating certain kinds of breast cancer. In this study, we opted to test TSC’s effect on several different breast cancer cell lines. Our results showed that TSC significantly inhibited the growth of ERα-positive breast cancer cell proliferation, down-regulated the expression of ERα, and blocked the estrogen signaling pathway. These findings suggest that TSC may inhibit ERα-positive breast cancer and mammary hyperplasia through suppressing ERα, making it a potentially viable clinical treatment option.

## Materials and Methods

TSC samples were provided by the Yunnan Institute of Meteria and Medica. From the TSC samples, we extracted the notoginseng with ethanol, and the other seven ingredients with water. All drug components were then condensed into solid powder. The resulting TSC powder was dissolved in sterilized ddH_2_O, and incubated in 50°C water bath for 1 h with shaking. Insoluble substances were removed by centrifuging at 3200 g for 20 mins. The resulting supernatant was filtered through 0.22 µm filter and stored at –20°C until further analysis.

### Cell culture

To test the effects on a variety of different breast cancer cells, we purchased human ERα-positive (MCF-7, T47D) and ERα-negative (HCC1937, SW527) breast cancer cell lines as well as immortalized breast epithelial cells (MCF10A and 184B5) from ATCC. All cells were maintained as monolayer cultures. MCF7 and T47D were cultured in MEM media supplemented with 10% FBS and 0.01 mg/ml insulin (Hyclone, Utah, USA). MCF10A and 184B5 cell lines were cultured in DMEM-F12 media supplemented with 2 mM L-glutamin, 1% Penicillin/Streptomycin, 200 ng/ml EGF, 100 ng/ml Cholera toxin, 0.01 mg/ml Insulin, 500 ng/ml Hydrocortisone, 5% Horse serum (Gibco, California, USA). HCC1937 was cultured in RPMI-1640 with 10% FBS. SW527 was cultured in DMEM media supplemented with 10% FBS. If necessary, MCF7 cells were cultured in MEM free of phenol red (Gibco) and T47D cells were cultured in RPMI-1640 free of phenol red with charcoal-stripped serum (Biological Industries, Kibbutz BeitHaemek, Israel).

### Sulforhodamine B colorimetric (SRB) assays

The SRB assay was performed to measure cell viability in 96-well plates. After treatment, cells were fixed with 10% trichloroacetic acid (TCA) and stained by SRB for 30 min. The excess dye was washed with 1% acetic acid. The protein-bound dye was dissolved in 10 mM Tris base solution for reading at OD_530_ using a microplate reader.

### Cell cycle analysis

Cell cycle distribution of MCF7 and T47D cells were determined using flow cytometry. Both MCF7 and T47D cells were treated with TSC (1 mg/ml) for 24 h in 6-well plates. The harvested cells were then washed twice with cold 1×PBS and treated with Cytofix/Cytoperm solution 250 µl (Becton Dickinson, NY, USA) in 4°C for 20 min. Following that, PI solution (100 µg/ml PI (Propidium Iodide #Z767557-1EA,Sigma, St Louis, MO), 100 µg/ml RNase A in PBS solution) was added to stain the cells for 30 min at 37°C.

### Cell proliferation assays

Cell proliferation was measured using the Edu Incorporation assay using Click-iT EdU Alexa Fluor 488 Imaging Kits (Invitrogen, Grand Island, NY). Cells were labeled by Edu (10 µM) for 6 h and fixed with 3.7% formaldehyde solution (soluble in PBS) for 15 min at room temperature, following which, 0.5% of Triton X-100 in PBS solution was added to the cells for 20 min at room temperature. The cells were then incubated with Click-iT reaction cocktail for 30 min and Hoechst 33342 solution (1∶2000 soluble in PBS) for 30 min at room temperature in dark conditions. Once the cells were ready, images were taken using a fluorescence microscope.

### Western blotting

Cells were washed three times in cold 1×PBS and collected with a lysis buffer (50 mM Tris-Cl pH 7.4, 150 mM NaCl, 1 mM EDTA, 1% Triton X-100). Equal amounts of protein lysate were used for western blot analyses with the indicated antibodies, which included: rabbit anti-ERα antibody (Santa Cruz Biotechnology, #sc-542, Santa Cruz, CA), rabbit anti-Cyclin D1 (Cell Signaling Technology, #2978s, Danvers, MA), Anti-β-actin Ab (Sigma, #A5411), anti-ERβ Ab (Novus Biologicals), and HRP coupled goat anti-mouse and HRP coupled goat anti-rabbit secondary antibodies (Santa Cruz Biotechnology). Images were collected using an ImageQuant LAS 4000 (GE HealthCare, Pittsburgh, PA).

### RT-PCR

Total RNA from MCF-7 or T47D cells was isolated using TRIzol (Invitrogen). cDNAs was synthesized with an iScript cDNA Synthesis Kit (Bio-Rad, CA) and subjected to qPCR with RT^2^ SYBR Green qPCR Mastermix (SABiosciences). Primer sequences for were as follows. ESR-1: 5′-CCACCAACCAGTGCACCATT-3′(F), 5′-GGTCTTTTCGTATCCCACCTTTC-3(R), CTSD: 5′-AGCTGGTGGACCAGAACATCT-3′(F), 5′-GGGTGACATTCAGGTAGGACA-3′(R), and Ps2: 5′-CATCGACGTCCCTCCAGAAGAG-3′(F), 5′-CTCTGGGACTAATCACCGTGCTG-3 (R).

### Knockdown of ERα

MCF7 cells were transfected with siRNAs against *ESR1* (5′-GGC ATG GAG CAT CTG TAC A-3′) and luciferase (5′-CTT ACG CTG AGT ACT TCG A-3′) control by lipofectamine 2000 (Life technologies). Twenty-four hours after siRNA transfection, the cells were treated with TSC for 12 h for WB or 48 h for the SRB assay.

### Dual luciferase assay

MCF7 and T47D cells were seeded in 24-well plates at 1×10^5^ cells per well. After 24 h, cells were transfected with ERα reporter plasmid (ERE)_2_-TK-Luc and internal control plasmid pRL-TK using Lipofectamine 2000 (Invitrogen). One day after transfection, the cells were serum starved for another day, then followed by TSC treatment. Luciferase activities were measured 24 h later by using the dual luciferase reporter assay system (Promega, Madison, WI).

### Statistical analysis

All experiments were repeated at least three times to ensure accuracy. Final, the values are expressed as mean ± standard deviation (S.D.) and analyzed by student t test. The level of significance: *p<0.05, **p<0.01.

## Results

### ER-α positive human breast cancer cells are more sensitive to TSC than ERα negative breast cancer cells

**Figure 1:**
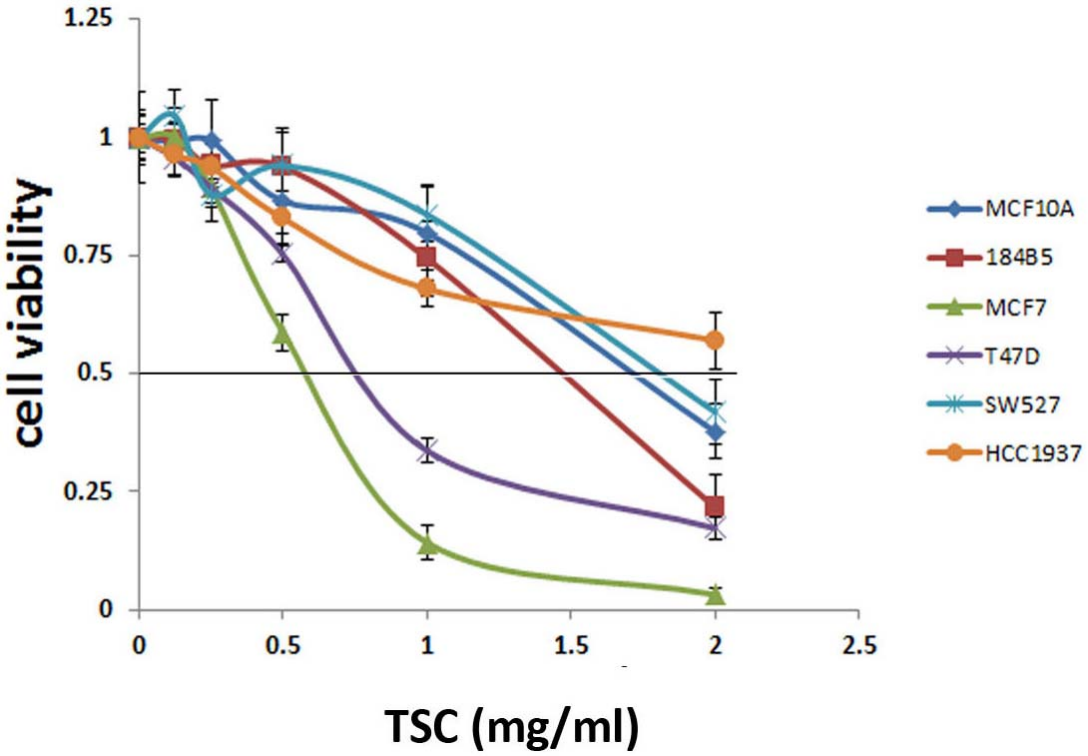
ERα-positive human breast cancer cells are more sensitive to TSC than ERα-negative breast cancer cells. Two immortalized mammary epithelial cell lines (MCF10A and 184B5), two ERα-positive breast cancer cell lines (MCF7 and T47D), and two ERα-negative breast cancer cell lines (SW527 and HCC1937) were treated with different concentrations of TSC (0.125, 0.25, 0.5, 1, 2 mg/ml) for 48 hours. Cell viability was measured by the SRB assay. ERα-positive breast cancer cell lines were found to be more sensitive to TSC than ERα-negative breast cell lines.

To test whether TSC inhibits growth of different types of breast cancer, we treated two immortalized mammary epithelial cell lines (MCF10A and 184B5), two ERα positive breast cancer cell lines (MCF7 and T47D), and two ERα negative breast cancer cell lines (SW527 and HCC1937) at varying concentrations of TSC (0.125, 0.25, 0.5, 1, 2 mg/ml) for 48 hours and then measured the cell viability via the SRB assay. TSC showed stronger growth inhibition toward ERα positive breast cancer cell lines (IC_50_ is 0.5–1 mg/ml) than the other four ERα negative breast cell lines (IC_50_>1.5 mg/ml) (Fig. 1). TSC’s observed inhibition of growth may be due to either increased cell death or decreased cell proliferation. To more fully explore the possible mechanism, we tested TSC to see if it induced apoptosis in MCF7 and T47D using Annexin V-FITC/7-AAD and cleaved PARP and caspase-7. After the cells were treated with 1 mg/ml TSC for 24 h, there was no significant increase of apoptosis as compared to the control group (data not shown).

### TSC inhibits cell proliferation through inducing the G1 cell cycle arrest

**Figure 2:**
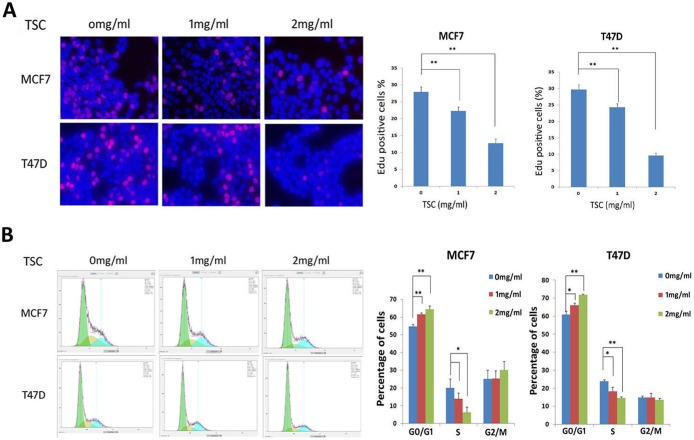
TSC inhibits cell proliferation through inducing the G1 cell cycle arrest. A. TSC significantly suppressed DNA synthesis in both MCF7 and T47D cell lines in a dosage dependent manner, as determined by the Edu incorporation assay. B. TSC caused G1 cell cycle arrest in both MCF7 and T47D cell lines in a dosage dependent manner as determined by flow cytometry. *: P<0.05; **: P<0.01.

To test whether TSC inhibits cell proliferation, we measured DNA synthesis using the EdU incorporation assay. TSC (1–2 mg/ml) significantly (P<0.01) decreased EdU-positive proliferative cell numbers in both MCF7 and T47D (Fig. 2A). Furthermore, we used PI staining to examine the cell cycle. As expected, TSC (1–2 mg/ml) significantly increased cells in the G1 phase and decreased cells in the S phase (Fig. 2B) in both MCF7 and T47D. The cell numbers in the G2/M phase showed no marked changes.

### TSC inhibits the expression of ERα and Cyclin D1

**Figure 3:**
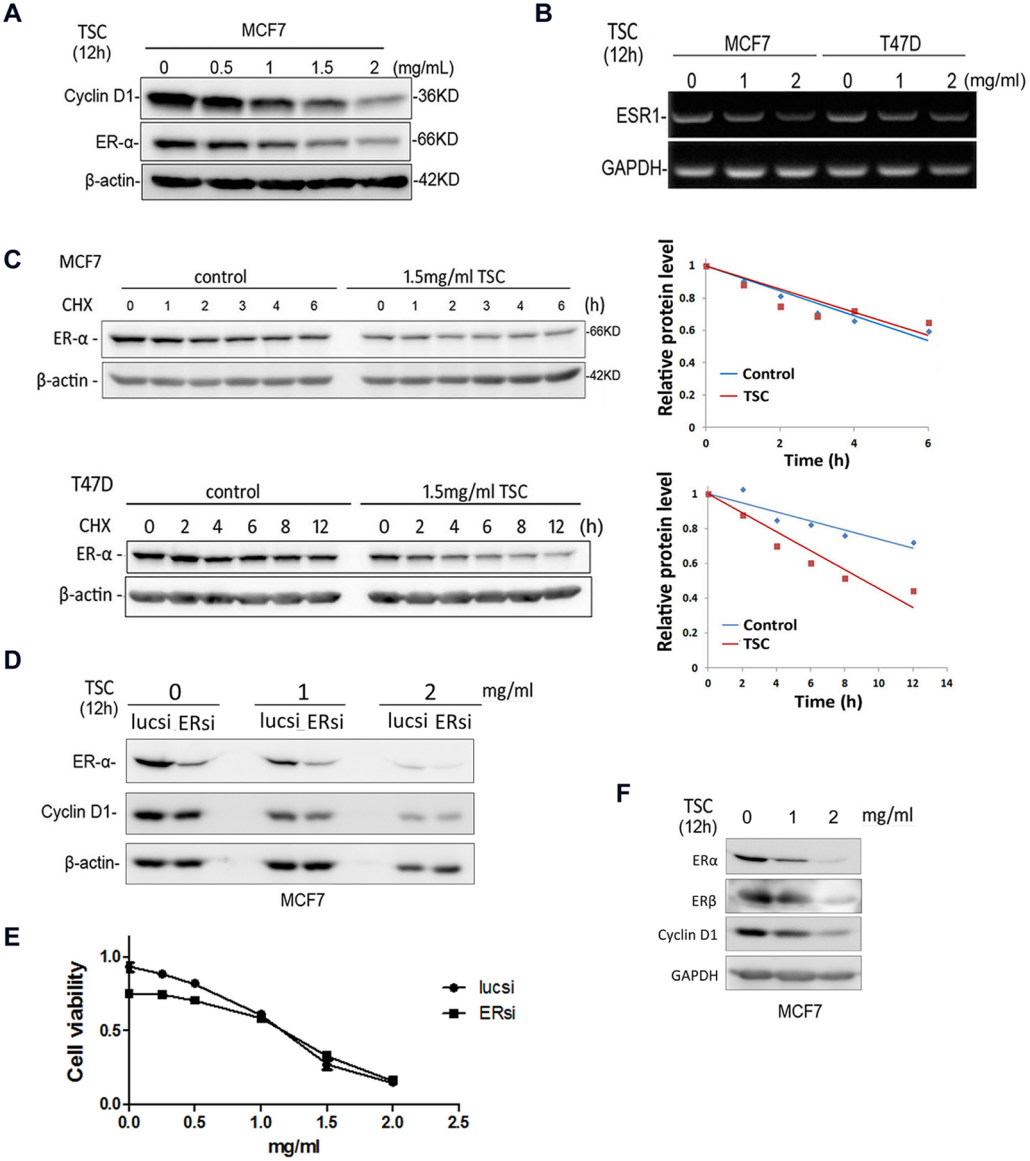
TSC inhibits the expression of ERα and Cyclin D1. A. TSC down-regulated the protein expression of ERα and Cyclin D1 in MCF7 in a dosage dependent manner. MCF7 cells were treated with different concentrations of TSC for 12 h. The protein expression levels of ERα and Cyclin D1 were examined using WB. B. The mRNA expression levels of *ESR1* are decreased by TSC in a dosage dependent manner in both MCF7 and T47D cell lines, as measured by RT-PCR. C. TSC promoted the protein degradation of ERα in T47D but not in MCF7, as detected by the cycloheximide (CHX) chase assay. Quantitative results are show on the right. D. Knockdown of ERα decreased the expression of Cyclin D1 in MCF7 and partially abrogated the effect of TSC on Cyclin D1 expression down-regulation. E. Knockdown of ERα decreased the cell viability of MCF7 and partially abrogated the effect of TSC on inhibiting MCF7. F. TSC down-regulated the protein expression of ERβ in MCF7. MCF7 cells were treated with different concentrations of TSC for 12 h. The protein expression levels of ERα, ERβ, and Cyclin D1 were examined by WB.

Since TSC predominately inhibits the growth of ERα positive breast cancer cells, we deduced that TSC may regulate the estrogen signaling pathway, which is well known to promote both cell proliferation and cell cycle. We treated MCF7 cells with different concentrations of TSC for 12 h and then examined the protein expression levels of ERα and Cyclin D1, which is a target of ERα. With the increase of TSC, the expression levels of ERα and Cyclin D1 gradually decreased (Fig. 3A). Interestingly, TSC also decreased the expression of ERβ in MCF7 (Fig. 3F); however, ERβ was not detected in T47D (data not shown). To further investigate precisely how TSC inhibits the ERα expression, we examined the *ESR1* mRNA levels using RT-PCR. The mRNA expression levels of *ESR1* were decreased in both MCF7 and T47D (Fig. 3B). We also examined the speed of ERα protein degradation using a cycloheximide chase assay, and interestingly, the degradation of the ERα protein was unchanged by TSC in MCF7 cells, but it was dramatically accelerated in T47D cells (Fig. 3C).

To further characterize the role of ERα in TSC-induced Cyclin D1 expression down-regulation and growth arrest in ERα-positive breast cancer cells, we knocked down ERα in MCF7 by siRNA, treated the cells with TSC, and examined the Cyclin D1 levels and cell viability. Depletion of endogenous ERα in MCF7 decreased the protein expression level of Cyclin D1 and cell viability (Fig. 3D–E). When endogenous ERα is silenced, TSC could not further reduce the protein expression level of Cyclin D1 and cell viability efficiently (Fig. 3D–E). These data suggest that TSC induces Cyclin D1 expression down-regulation and growth arrest partially through inhibiting the expression of ERα.

### TSC inhibits the estrogen signal pathway

**Figure 4:**
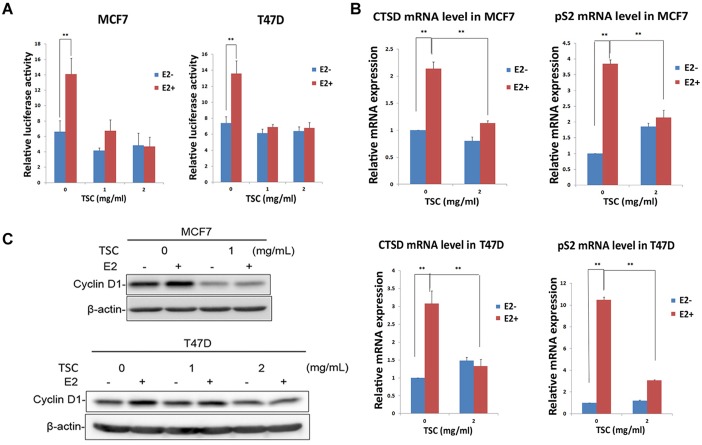
TSC inhibits the estrogen signal pathway. A. TSC inhibited the transcriptional activation of ERα in response to E2 in both MCF7 and T47D, as measured by the ERE luciferase reporter assay. **, P<0.01. B. TSC blocked the E2-induced *CTSD* and *pS2* mRNA expression in both MCF7 and T47D, as measured by real-time PCR. **, P<0.01. C. TSC blocked the induction of Cyclin D1 by E2 in MCF7 and T47D cells. The E2 concentration was 10 nM.

Given that TSC down-regulated the expression of ERα and its downstream target gene Cyclin D1 in regular media, we suspected that TSC may suppress breast cancer cell growth by blocking the pro-proliferative function of estrogen. We first used an ERE reporter plasmid to test whether TSC inhibits the transcriptional activation of ERα. As shown in Fig. 4A, E2 stimulated the ERE luciferase reporter activity for about 2 folds while TSC (1–2 mg/ml) almost completely blocked the E2-induced luciferase reporter activity increase in both MCF7 and T47D in the media without estrogen.

To further confirm whether TSC inhibits the estrogen signaling pathway, we measured the mRNA expression levels of two important E2/ERα target genes: *CTSD* and *pS2* (Fig. 4B). qRT-PCR showed that the mRNA levels *CTSD* and *pS2* were significantly increased in MCF7 and T47D cells after E2 treatment in the media without estrogen. TSC (2 mg/ml) almost completely blocked the E2-induced *CTSD* and *pS2* mRNA expression (Fig. 4B). Consistently, TSC (1–2 mg/ml) also blocked the induction of Cyclin D1 by E2 in MCF7 and T47D cells (Fig. 4C).

### TSC inhibits the E2-induced cell cycle progression and cell proliferation

**Figure 5:**
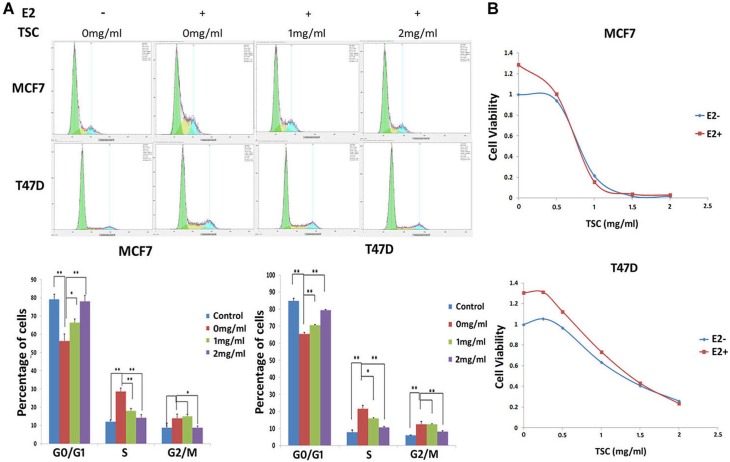
TSC inhibits the E2-induced cell cycle progression and cell proliferation. **A**. TSC blocked the E2-induced G1/S cell cycle progression in both MCF7 and T47D, as measured by flow cytometry. *, P<0.05; **, P<0.01. **B**. TSC significantly blocked the E2-induced cell growth in both MCF7 and T47D cell lines, as measured by the SRB assay.

Estrogen is well established to promote cell cycle progression and stimulate cell proliferation through ERα. Since TSC suppresses the expression of ERα and its downstream target genes, it stands to reason that it may also inhibit estrogen-induced cell cycle progression and cell proliferation. Our results showed that in both MCF7 and T47D cell lines, E2 significantly decreased the cell population in the G1 phase and increased the cell population in the S phase in the media without estrogen (Fig. 5A). TSC (1–2 mg/ml) blocked the E2-induced G1/S cell cycle progression in a dosage dependent manner. In terms of cell growth, E2 indeed accelerated the growth of MCF7 and T47D, but TSC also significantly blocked the E2-induced cell growth in a dosage dependent manner in both MCF7 and T47D (Fig. 5B).

## Discussion

Breast cancer and mammary hyperplasia are both common breast diseases in women in which estrogen signaling pathways play important roles. Previous studies have shown that the ERα antagonist tamoxifen is effective in the treatment of ERα-positive breast cancer and mammary hyperplasia [10], [14], [15]. In this study, we demonstrated that TSC, a traditional Chinese medicine that has likewise been effectively used to treat mammary hyperplasia, predominately inhibits ERα-positive breast cancer cell growth, potentially by down-regulating of ERα expression, thus making ERα-positive breast epithelial cells less sensitive to estrogen-induced cell proliferation.

Our results showed that in MCF7 and T47D, TSC actually reduced *ESR1* mRNA. While in MCF7 cells, the degradation of ERα does not change significantly, but TSC enhances degradation of ERα in T47D cells (Fig. 3). Interestingly, TSC also decreased the expression of ERβ in MCF7 (Fig. 3F); however, ERβ was not expressed in T47D [16]. These differences may be due to the heterogeneity of cancer cell lines, though clearly more follow-up studies are needed to verify this supposition. Likewise, the composition of TSC itself is extremely complex and multiple targets are expected for TSC. We found that one ingredient by itself, notoginseng, can down-regulate the expression of ERα in MCF7 and T47D; however, notoginseng alone can differentially regulate the expression of ERα target genes *CTSD* and *pS2* (data not shown). These results suggest that components from other Chinese medicines that comprise TSC aside from notoginseng play important roles in regulating the expression of ERα target genes. TSC also suppress ERα-negative breast epithelial cell growth in spite of less potency, suggesting that TSC also functions through other mechanisms aside from just inhibiting ERα. Since the effective compounds of TSC are unknown at present time, we do not know if the effective concentration of TSC *in* *vitro* can be reached *in* *vivo*. Again, more detailed studies are needed to identify the other functional components for TSC.

In this study, we demonstrated that TSC caused G1 cell cycle arrest instead of inducing apoptosis in two ERα-positive breast cancer cell lines. The G1/S phase transition is mainly driven through cyclin dependent kinase CDK4 and CDK6. The ERα target gene *CCND1/Cyclin D1* can activate CDK4/6 and drive the G1/S transition. As expected, TSC inhibits the expression of CyclinD1 and estrogen-induced CyclinD1 (Fig. 3). Since Cyclin D1 is also regulated by many other signaling pathways, we cannot completely exclude the possibility that TSC regulates Cyclin D1 expression through other signaling pathways. Actually, it is unknown whether TSC inhibits the expression of Cyclin D1 in an ERα-dependent manner because TSC repressed cell proliferation in both ERα-negative and ERα-positive breast cancer cell lines. Moreover, it is unknown whether TSC inhibits breast cancer cell proliferation through the down-regulation of Cyclin D1. TSC also inhibited the expression of two other ERα target genes *CTSD* and *pS2* (Fig. 4). It then seems likely that TSC suppresses the cell cycle progression through inhibiting the expression of multiple ERα target genes.

On the whole, ERα positive breast cancer accounts for about 70% of total breast cancers. To date, a variety of drugs used to inhibit the estrogen signaling pathway and treat ERα positive breast cancers–e.g., tamoxifen, fulvestrant, and aromatase inhibitor letrozole–have been approved. Tamoxifen does not inhibit the expression of ERα in both MCF7 and T47D [17] although it inhibits the expression of CyclinD1 in T47D [18]. In estrogen-maintained MCF7 cells, tamoxifen increased levels of ERα. By contrast, in estrogen-deprived MCF7 cells, long-term tamoxifen exposure decreased ERα levels [19]. Fulvestrant induces the degradation of ERα and the expression of pS2 in MCF7 in the presence or absence of estrogen [19], [20]. TSC could also inhibit the estrogen signaling pathway and suppress the MCF7 and T47D cell growth *in* *vitro*. More poignantly, TSC may actually be a viable treatment option for ERα-positive breast cancer patients. Obviously, xenograft models will be required to test whether TSC inhibits the growth of ERα-positive breast tumors *in* *vivo*, and establishing rat or rabbit models of mammary hyperplasia on which to test the therapeutic effects of TSC and its underlying functional mechanisms may also prove worthwhile. Nevertheless, in the meantime, given that TSC is currently approved by the Chinese FDA, it should be relatively simple to set up phase IV clinical trials and test the therapeutic benefits suggested by this study as well as the previous reports.

In summary, we found that the Tongshu Capsule inhibits the expression of ERα and its downstream target genes, subsequently decreasing estrogen-induced cell cycle progression and proliferation in ERα-positive breast cancer cell lines. Our findings further revealed one possible mechanism by which TSC achieved its therapeutic benefits among patients with mammary hyperplasia, implying that that TSC could prove a viable alternative in treating ERα-positive breast cancer patients.

## References

[1] Santen RJ, Mansel R (2005) Benign breast disorders. New England Journal of Medicine 353: 275–285. 16034013 10.1056/NEJMra035692

[2] Allegra JC, Lippman ME, Green L, Barlock A, Simon R, et al (1979) Estrogen receptor values in patients with benign breast disease. Cancer 44: 228–231. 455247 10.1002/1097-0142(197907)44:1<228::aid-cncr2820440137>3.0.co;2-0

[3] Sasaki Y, Miki Y, Hirakawa H, Onodera Y, Takagi K, et al (2010) Immunolocalization of estrogen-producing and metabolizing enzymes in benign breast disease: Comparison with normal breast and breast carcinoma. Cancer science 101: 2286–2292. 20682005 10.1111/j.1349-7006.2010.01673.x PMC11159500

[4] Lagiou P, Samoli E, Lagiou A, Zourna P, Barbouni A, et al (2013) A comparison of hormonal profiles between breast cancer and benign breast disease: a case–control study. Annals of Oncology. 10.1093/annonc/mdt207 PMC3784331 23723293

[5] Key T, Appleby P, Barnes I, Reeves G (2002) Endogenous sex hormones and breast cancer in postmenopausal women: reanalysis of nine prospective studies. Journal of the National Cancer Institute 94: 606–616. 11959894 10.1093/jnci/94.8.606

[6] Key T, Appleby P, Reeves G, Roddam A, Dorgan J, et al (2003) Body mass index, serum sex hormones, and breast cancer risk in postmenopausal women. Journal of the National Cancer Institute 95: 1218–1226. 12928347 10.1093/jnci/djg022

[7] Hall JM, Couse JF, Korach KS (2001) The multifaceted mechanisms of estradiol and estrogen receptor signaling. Journal of Biological Chemistry 276: 36869–36872. 11459850 10.1074/jbc.R100029200

[8] Foster JS, Wimalasena J (1996) Estrogen regulates activity of cyclin-dependent kinases and retinoblastoma protein phosphorylation in breast cancer cells. Molecular Endocrinology 10: 488–498. 8732680 10.1210/mend.10.5.8732680

[9] Altucci L, Addeo R, Cicatiello L, Dauvois S, Parker MG, et al (1996) 17beta-Estradiol induces cyclin D1 gene transcription, p36D1-p34cdk4 complex activation and p105Rb phosphorylation during mitogenic stimulation of G (1)-arrested human breast cancer cells. Oncogene 12: 2315–2324. 8649771

[10] Fisher B, Costantino J, Redmond C, Poisson R, Bowman D, et al (1989) A randomized clinical trial evaluating tamoxifen in the treatment of patients with node-negative breast cancer who have estrogen-receptor–positive tumors. New England Journal of Medicine 320: 479–484. 2644532 10.1056/NEJM198902233200802

[11] Sabbah M, Courilleau D, Mester J, Redeuilh G (1999) Estrogen induction of the cyclin D1 promoter: involvement of a cAMP response-like element. Proceedings of the National Academy of Sciences 96: 11217–11222. 10.1073/pnas.96.20.11217 PMC18014 10500157

[12] Li X, Zhang J, Yu L (2006) Clinical analysis: Tongshu capsule treats 60 cases of mammary hyperplasia. Guide of China Medicine 4.

[13] Chen J (2011) Curative effect observation on Tonshu capsule combines with vitamin E to treat mammary hyperplasia. Hebei Medical Journal 33: 623–624.

[14] Kontostolis E, Stefanidis K, Navrozoglou I, Lolis D (1997) Comparison of tamoxifen with danazol for treatment of cyclical mastalgia. Gynecological Endocrinology 11: 393–397. 9476088 10.3109/09513599709152566

[15] Maddox PR, Mansel MRE (1989) Management of breast pain and nodularity. World journal of surgery 13: 699–705. 2696222 10.1007/BF01658417

[16] Cheng L, Li J, Han Y, Lin J, Niu C, et al (2012) PES1 promotes breast cancer by differentially regulating ERalpha and ERbeta. J Clin Invest 122: 2857–2870. 22820289 10.1172/JCI62676 PMC3408741

[17] Sartippour MR, Pietras R, Marquez-Garban DC, Chen HW, Heber D, et al (2006) The combination of green tea and tamoxifen is effective against breast cancer. Carcinogenesis 27: 2424–2433. 16785249 10.1093/carcin/bgl066

[18] Chu I, Blackwell K, Chen S, Slingerland J (2005) The dual ErbB1/ErbB2 inhibitor, lapatinib (GW572016), cooperates with tamoxifen to inhibit both cell proliferation- and estrogen-dependent gene expression in antiestrogen-resistant breast cancer. Cancer Res 65: 18–25. 15665275

[19] Shaw LE, Sadler AJ, Pugazhendhi D, Darbre PD (2006) Changes in oestrogen receptor-alpha and -beta during progression to acquired resistance to tamoxifen and fulvestrant (Faslodex, ICI 182, 780) in MCF7 human breast cancer cells. J Steroid Biochem Mol Biol 99: 19–32. 16533599 10.1016/j.jsbmb.2005.11.005

[20] Callige M, Kieffer I, Richard-Foy H (2005) CSN5/Jab1 is involved in ligand-dependent degradation of estrogen receptor {alpha} by the proteasome. Mol Cell Biol 25: 4349–4358. 15899841 10.1128/MCB.25.11.4349-4358.2005 PMC1140630

